# The regulatory effects of (p)ppGpp and indole on cAMP synthesis in Escherichia coli cells

**DOI:** 10.18699/VJGB-24-03

**Published:** 2024-02

**Authors:** N.M. Kashevarova, E.A. Khaova, A.G. Tkachenko

**Affiliations:** Institute of Ecology and Genetics of Microorganisms of the Ural Branch of the Russian Academy of Sciences, Perm, Russia; Institute of Ecology and Genetics of Microorganisms of the Ural Branch of the Russian Academy of Sciences, Perm, Russia; Institute of Ecology and Genetics of Microorganisms of the Ural Branch of the Russian Academy of Sciences, Perm, Russia

**Keywords:** Escherichia coli, signaling molecules, cAMP, (p)ppGpp, indole, glucose, tryptophan, Escherichia coli, сигнальные молекулы, цАМФ, (p)ppGpp, индол, глюкоза, триптофан

## Abstract

Bacterial stress adaptive response is formed due to changes in the cell gene expression profile in response to alterations in environmental conditions through the functioning of regulatory networks. The mutual influence of network signaling molecules represented by cells’ natural metabolites, including indole and second messengers (p) ppGpp and cAMP, is hitherto not well understood, being the aim of this study. E. coli parent strain BW25141 ((p) ppGpp+) and deletion knockout BW25141ΔrelAΔspoT which is unable to synthesize (p)ppGpp ((p)ppGpp0) were cultivated in M9 medium supplemented with different glucose concentrations (5.6 and 22.2 mM) in the presence of tryptophan as a substrate for indole synthesis and in its absence. The glucose content was determined with the glucose oxidase method; the indole content, by means of HPLC; and the cAMP concentration, by ELISA. The onset of an increase in initially low intracellular cAMP content coincided with the depletion of glucose in the medium. Maximum cAMP accumulation in the cells was proportional to the concentration of initially added glucose. At the same time, the (p) ppGpp0 mutant showed a decrease in maximum cAMP levels compared to the (p)ppGpp+ parent, which was the most pronounced in the medium with 22.2 mM glucose. So, (p)ppGpp was able to positively regulate cAMP formation. The promoter of the tryptophanase operon responsible for indole biosynthesis is known to be under the positive control of catabolic repression. Therefore, in the cells of the (p)ppGpp+ strain grown in the tryptophan-free medium that were characterized by a low rate of spontaneous indole formation, its synthesis significantly increased in response to the rising cAMP level just after glucose depletion. However, this was not observed in the (p)ppGpp0 mutant cells with reduced cAMP accumulation. When tryptophan was added to the medium, both of these strains demonstrated high indole production, which was accompanied by a decrease in cAMP accumulation compared to the tryptophan-free control. Thus, under glucose depletion, (p)ppGpp can positively regulate the accumulation of both cAMP and indole, while the latter, in its turn, has a negative effect on cAMP formation.

## Abstract (ru)

Адаптивный ответ бактерий на стрессорное воздействие условий среды формируется за счет изменения генно-экспрессионного профиля клетки посредством функционирования регуляторных сетей. Сигналами в них выступают естественные метаболиты клетки, в частности индол, а также вторичные мессенджеры (p) ppGpp и цАМФ, взаимное влияние которых недостаточно изучено и представляет собой цель данного исследования. Родительский штамм E. coli BW25141 ((p)ppGpp+) и нокаут BW25141ΔrelAΔspoT ((p)ppGpp0), не способный синтезировать (p)ppGpp, культивировали на среде М9 с различным содержанием глюкозы (5.6 и 22.2 мМ), в присутствии триптофана в качестве субстрата для синтеза индола и в его отсутствие. Содержание глюкозы измеряли глюкозооксидазным методом, уровень индола – при помощи ВЭЖХ, концентрацию цАМФ – методом ELISA. Начало возрастания исходно низкого внутриклеточного содержания цАМФ совпадало с исчерпанием глюкозы в среде. Максимальный уровень накопления цАМФ в клетках был пропорционален концентрации исходно добавленной глюкозы. При этом (p)ppGpp0 мутант демонстрировал снижение максимального уровня цАМФ по сравнению с (p)ppGpp+ родителем, наиболее выраженное на среде с 22.2 мМ глюкозы. Таким образом, (p)ppGpp положительно регулирует образование цАМФ. Известно, что промотор триптофаназного оперона, ответственного за биосинтез индола, находится под положительным контролем механизма катаболитной репрессии. Поэтому в клетках (p)ppGpp+ штамма в условиях низкой скорости спонтанного образования индола на бестриптофановой среде значительно усиливался его синтез в ответ на возрастание уровня цАМФ при исчерпании глюкозы, чего не наблюдалось у (p)ppGpp0 мутанта с пониженным накоплением цАМФ. При добавке триптофана оба штамма демонстрировали высокую продукцию индола, что сопровождалось снижением уровня накопления цАМФ по сравнению с бестриптофановым контролем. Таким образом, (p)ppGpp при исчерпании глюкозы положительно регулирует накопление цАМФ и индола, который, в свою очередь, снижает образование цАМФ.

## Introduction

The adaptation of bacteria to stress is based on a subtle tuning of regulatory networks by the changes in gene expression and enzyme activity. Furthermore, the metabolic products of bacterial cells, such as indole and second messengers ( p)ppGpp and cAMP, are regulatory signals. The interaction of the metabolic pathways of these signaling molecules ensures the adaptation of bacteria to various changes in the environment, which is of great scientific interest

Among the currently known nucleotide second messengers, cAMP (3′,5′-cyclic adenosine monophosphate) and (p)ppGpp (guanosine tetra(penta)phosphate) were the first to be studied in most detail in the model organism Escherichia coli. They act as intracellular “secondary” signals, transducing information about the actual microenvironment of bacterial cells by transforming the extracellular “primary” signals into a cascade of intracellular physiological changes necessary to support important biological functions (Hengge et al., 2019).

cAMP is synthesized by the enzyme adenylate cyclase, encoded by the cyaA gene, that catalyzes the cyclization reaction of adenosine triphosphate (ATP) to form cAMP and inorganic pyrophosphate (Botsford, Harman, 1992) and is cleaved to form AMP by means of phosphodiesterases CpdA (Imamura et al., 1996) and DosP (Yoshimura-Suzuki et al., 2005). The global regulator cAMP plays an important role in many biological processes, including the regulation of growth, differentiation and general cell metabolism, division, starvation, anaerobiosis, carbon metabolism, stress reactions (Gosset et al., 2004), osmoregulation (Balsalobre et al., 2006), and quorum sensing (Zhou et al., 2008). Transcriptomic analysis has shown that more than 200 operons are under direct or indirect control of cAMP (Gutierrez-Ríos et al., 2007). cAMP in complex with the cAMP receptor protein (CRP) (Rickenberg, 1974) regulates the transcription of several hundred genes, some of which are involved in catabolic processes (Botsford, Harman, 1992), and thus participates in multiple regulatory networks.

Another well-known second messenger, (p)ppGpp, induces one of the main bacterial adaptive mechanisms – the stringent response (Potrykus, Cashel, 2008; Hauryliuk et al., 2015), affecting various aspects of bacterial physiology, such as persistence, virulence, biofilm formation, and others (Dalebroux et al., 2010; Maisonneuve, Gerdes, 2014). Since the (p)ppGpp alarmone is a global regulator that controls the expression of about 1,400 genes (Traxler et al., 2008), its main function is to regulate the growth and survival of bacterial cells in response to nutrient deficiency and various stress factors (Hengge et al., 2019). Elevated levels of (p)ppGpp delay bacterial growth by suppressing the production of RNA, DNA, and proteins (Mechold et al., 2013). In E. coli cells, (p)ppGpp levels are determined by the balance between two alarmone synthetases RelA/SpoT, the homologues of (RSH) protein family – RelA with only synthesizing activity and the bifunctional enzyme SpoT, which exhibits both (p)ppGpp synthetase and hydrolase activities. RelA synthetase is activated upon binding to ribosomes during amino acid starvation, when uncharged tRNAs bind to the A site of the ribosome. SpoT-dependent accumulation of (p)ppGpp in bacterial cells occurs when there is a lack of carbon sources, fatty acids, iron, nitrogen, phosphates, as well as under oxidative stress, etc. (Arenz et al., 2016). In E. coli cells, (p)ppGpp regulates gene transcription by changing the activity of RNA polymerase via direct interaction with the enzyme or indirectly, reducing the cellular pool of guanosine triphosphate (GTP) and, accordingly, the ATP/ GTP ratio as a result of the consumption of GTP for (p)ppGpp synthesis (Dalebroux, Swanson, 2012; Hauryliuk et al., 2015).

Indole, a signaling molecule of stationary phase bacterial cells, also takes part in a signal transduction of changes in conditions of bacterial microenvironment. Indole production by E. coli cells, discovered in the early 20th century, has previously been used as a diagnostic marker to differentiate E. coli from other enteric bacteria. However, as it is now known, the ability of bacteria to synthesize indole is widespread among different species of microorganisms. It is produced by at least 27 genera of bacteria capable of synthesizing the enzyme tryptophanase TnaA, which breaks down tryptophan to produce indole, pyruvate and ammonium (Lee et al., 2007). Indole is imported into the cell mainly through Mtr permease (Yanofsky et al., 1991) and is exported from the cell using the multidrug efflux pump AcrEF (Kawamura-Sato et al., 1999). In E. coli cells, indole regulates such important cellular processes as biofilm formation (Lee et al., 2007), persistence (Kwan et al., 2015), the development of multidrug resistance (Hirakawa et al., 2005), changes in plasmid stability (Chant, Summers, 2007), motility (Bansal et al., 2007), and the ability to survive in mixed bacterial cultures (Chu et al., 2012).

Regulatory networks including the signaling molecules cAMP, (p)ppGpp and indole closely interact in bacterial cells to transduce intracellular or environmental signals for triggering a cascade of biochemical reactions leading to changes in cell gene expression profile and metabolism. One of the regulators of indole synthesis is cAMP, which, as part of the cAMP-CRP complex, positively regulates the transcription of tryptophanase operon responsible for indole production (Stewart, Yanofsky, 1985). We have previously shown that indole formation in E. coli is a (p)ppGpp-dependent process (Kashevarova et al., 2022). Decreased cAMP levels as a result of degradation by phosphodiesterases reduce indole production, increasing persistence (Kwan et al., 2015). At the same time, the works of other researchers showed that exogenous addition of cAMP, with the participation of the cAMP-CRP regulatory complex controlling the expression of RelA synthetase, stimulated its expression and resulted in an increase in (p)ppGpp production and persistence. Moreover, the activity of the cAMP-CRP complex and the cAMP-CRP-controlled expression of RelA increased upon glucose depletion in both normal and persistent cells (Amato et al., 2013). Thus, regulatory signals cAMP and indole (Kwan et al., 2015), along with (p)ppGpp (Wood et al., 2013), are involved in multiple persistence pathways.

The purpose of our study is to study the effect of (p)ppGpp and indole on the formation of cAMP under conditions of depletion of the carbon substrate glucose.

## Materials and methods

Strains and culture conditions. The objects of the study were the E. coli BW25141 parent strain (WT, (p)ppGpp+) (Datsenko, Wanner, 2000) and the BW25141ΔrelAΔspoT mutant ((p)ppGpp0) (laboratory collection). Cultures stored on Luria–Bertani (LB) agar (Sigma, USA) were cultivated for 5 hours at 37 °C in tubes with 5 mL of LB broth (Sigma, USA). Then, the cells were transferred to Erlenmeyer flasks with minimal M9 medium (50 mL) containing 5.6 mM (0.1 %) or 22.2 mM (0.4 %) glucose and cultivated at 120 rpm for 18 hours in a thermostated shaker GFL 1092 (GFL, Germany). Overnight cultures were diluted in 50 mL of M9 medium with appropriate concentrations of glucose to OD600 = 0.2. Tryptophan (2 mM) (AppliChem, Germany) was added to some of the flasks, keeping the others as controls, and then cultivated under the same conditions for 168 hours. The optical density of the cultures was measured by the absorbance value at 600 nm on a UV-1650PC spectrophotometer (Shimadzu, Japan).

Gene engineering. The BW25141ΔrelAΔspoT deletion mutant was constructed using the FLP/FRT site-specific recombination system (Datsenko, Wanner, 2000).

Glucose content in the medium was determined by the glucose oxidase method using a Glucose-Vital kit (Vital Development Corporation, Russia). 5 μL of the samples were taken from the exponentially growing cultures with a time interval of one hour, and then once a day, and incubated with 1 mL of the kit reagent at room temperature for 15 minutes. When glucose is oxidized by glucose oxidase under the atmospheric pressure, an equimolar amount of hydrogen peroxide is formed that, in its turn, oxidizes chromogenic substrates by means of peroxidase to form a colored product proportionally to glucose concentration that was measured photometrically at 510 nm on the UV-1650PC spectrophotometer (Shimadzu, Japan).

Determination of indole. The concentration of indole in the medium was measured by high-performance liquid chromatography (Kim et al., 2013), with minor modifications, on an LC-20A chromatograph with an SPD-M20A spectrophotometric detector (Shimadzu, Japan), Luna C18 column (250×4.6 mm, 5 μm), SecurityGuard C18 pre-column (4×3 mm) (Phenomenex, USA). To determine extracellular indole, the samples were taken from the exponential phase cultures with a time interval of 2 hours and once a day from the stationary phase ones. Cells were pelleted by centrifugation. Supernatant samples in a volume of 20 μL were analyzed at a mobile phase flow rate of 1 mL/min (a mixture of acetonitrile (Kriochrome, Russia) and acetic acid in a 1:1 ratio) and +25 °C with detection at a wavelength of 280 nm. To calculate the indole content in the sample, the external standard method was used based on a previously constructed calibration curve.

Measurement of cAMP concentration. Quantitative determination of intracellular cAMP concentration was carried out by the competitive enzyme-linked immunosorbent assay (ELISA). 1.5 mL of the test culture was centrifuged at 12,000 rpm at +4 °C for 5 minutes. The pellet was resuspended in 300 μL of 0.1N HCl, kept for 5 minutes at +95 °C, followed by destruction of cells using a CPX-130 ultrasonic disintegrator (Cole-Parmer, USA), using a probe with a diameter of 6 mm (3 cycles for 30 seconds at an amplitude of 40 %). Cell residues were precipitated by centrifugation for 15 minutes. cAMP concentration was determined in the resulting supernatant after neutralization with 2 M Na2CO3 according to the manufacturer’s instruction (RayBio® cAMP Enzyme Immunoassay). Experiments were carried out with at least two independent cultures. The data obtained were normalized to intracellular volume based on absolutely dry cell biomass

Statistical analysis. The results obtained were processed statistically using the standard software package Statistica 6.0 (StatSoft Inc., USA). On the graphs, the means (at least three experiments) and the standard deviations are represented. The significance of differences was assessed using Student’s t-test ( p ≤ 0.05).

## Results

In the present work, the dynamics of glucose depletion and the formation of cAMP and indole in periodic cultures of the E. coli BW25141 wild-type strain and the ΔrelAΔspoT deletion mutant grown in M9 mineral medium with different contents of initially added glucose (5.6 and 22.2 mM) were studied. Overproduction of indole was induced by exogenous addition of the amino acid tryptophan (2 mM), a precursor of indole synthesis, in order to study its effect on the activity of metabolic processes, in particular, the consumption rate of carbon substrate (glucose) and cAMP formation.

**Fig. 1.**
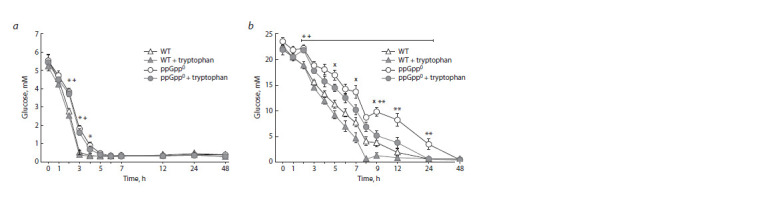
Dynamics of glucose consumption in cultures of the parental strain (WT) and the (p)ppGpp0 mutant in the medium without tryptophan and with the addition of 2 mM tryptophan at 5.6 mM (a) and 22.2 mM glucose (b). * Statistically significant difference between the (p)ppGpp0 mutant and the (p)ppGpp+ strain in the medium without tryptophan; + statistically significant difference between the (p)ppGpp0 mutant and the (p)ppGpp+ strain in the medium with the addition of 2 mM tryptophan; x statistically significant difference between the (p)ppGpp+ strain supplemented with 2 mM tryptophan and the (p)ppGpp+ strain in the tryptophan-free medium; ** statistically significant difference between the (p)ppGpp0 mutant in the medium with 2 mM tryptophan and the (p)ppGpp0 one in the tryptophan-free medium ( р ≤ 0.05).

When studying the glucose consumption in the strain periodic cultures, it was found that at an initially low concentration of glucose (5.6 mM), substrate depletion in the cells of both strains studied occurred in the first hours of cultivation (Fig. 1, a). Moreover, in the culture of the parental strain, glucose was completely consumed after 3 hours of growth, while (p)ppGpp absence in the cells of the ΔrelAΔspoT mutant led to a delay in substrate consumption by 2 hours as compared to the wild-type strain. At the same time, the presence of tryptophan in the medium had no effect in both cases.

In the culture of parent strain with high initial glucose concentration (22.2 mM), it was consumed for 12 hours after the cells were inoculated to a fresh nutrient medium (Fig. 1, b). The double ΔrelAΔspoT mutation, as well as at the low glucose concentration (5.6 mM), led to a slowing down of substrate utilization rate, so that glucose was consumed for more than 24 hours. However, the addition of tryptophan to the medium caused an acceleration of glucose consumption for more than 4 hours in the parental strain and for 24 hours in the (p)ppGpp0 mutant.

**Fig. 2.**
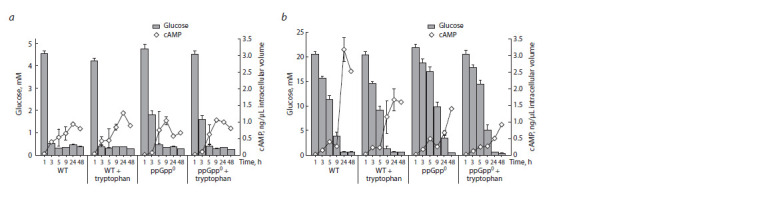
Glucose consumption (histogram/columns) and cAMP formation (graph/diamonds) in cultures of the parent strain and the (p)ppGpp0 mutant in the medium without tryptophan and with the addition of 2 mM tryptophan at content of 5.6 mM (a) and 22.2 mM glucose (b).

The onset of an increase in the initially low intracellular level of cAMP coincided with the depletion of glucose in the medium. Moreover, in cultures grown in the medium with 5.6 mM glucose, the increase in cAMP concentration occurred earlier than in those grown in the medium with the addition of 22.2 mM glucose (Fig. 2). At low glucose content in the medium, the (p)ppGpp+ strain showed rapid depletion of glucose for the first 1–3 hours, which was accompanied by an active accumulation of cAMP during the same period (see Fig. 2, a). However, the double ΔrelAΔspoT deletion led to a sharper increase in cAMP levels starting at 5 hours, which was not observed in the medium containing 22.2 mM glucose. By the end of the observed period of the first day of cultivation (9 hours of growth), higher values of cAMP accumulation were demonstrated by the mutant at 5.6 mM glucose, which was approximately 2 times higher than the cAMP level at 22.2 mM glucose.

**Fig. 3.**
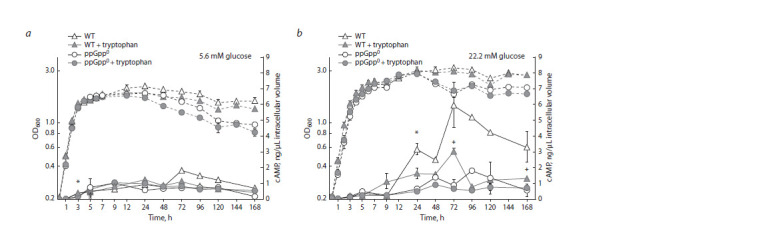
Growth curves (dashed lines) and cAMP formation (solid lines) in the cultures of parent and mutant strains in the medium without tryptophan (open symbols) and with the addition of 2 mM tryptophan (filled symbols) at a content of 5.6 mM (a) and 22.2 mM glucose (b) in the medium. * Statistically significant difference between the (p)ppGpp0 mutant and the (p)ppGpp+ strain in the medium without tryptophan; + statistically significant difference between the (p)ppGpp0 mutant and the (p)ppGpp+ strain at the supplementation of 2 mM tryptophan

In stationary cultures, a high concentration of initially added glucose (22.2 mM) in the medium led to higher levels of cAMP accumulation than at 5.6 mM glucose; however, in the case of the deletion mutant, these differences were less pronounced (Fig. 3).

The maximum values of cAMP accumulation in cells were proportional to the concentration of added glucose. At the same time, the (p)ppGpp0 knockout demonstrated a decrease in the maximum level of cAMP compared to the WT strain that was the most pronounced in the medium with 22.2 mM glucose. Maximum accumulation of cAMP at 5.6 mM glucose content in the mutant strain was achieved at 9 hours of cultivation with a subsequent decrease, whereas in the (p)ppGpp+ parent, by 72 hours in the tryptophan-free medium and 24 hours in the one supplemented with tryptophan (see Fig. 3, a). At high glucose content (22.2 mM), the maximum cAMP values in the WT strain were observed at 72 hours of growth both in the tryptophan-free medium and in the presence of the indole precursor (see Fig. 3, b). At the same time, the cAMP level in the (p)ppGpp0 knockout changed slightly throughout the entire cultivation period, especially in the medium supplemented with tryptophan, and was up to 6 times lower as compared to the WT strain. Thus, the absence of (p)ppGpp in the cells led to a decrease in cAMP production.

**Fig. 4.**
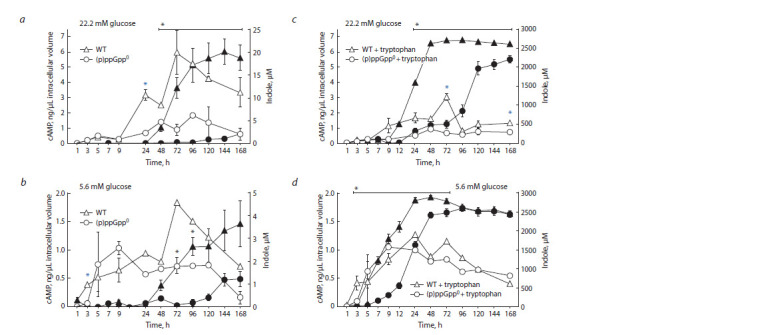
Indole production (filled symbols) and cAMP formation (open symbols) in cultures of the parent strain (WT) and the (p)ppGpp0 mutant in the medium without tryptophan (a, b) and with the addition of 2 mM tryptophan (c, d) at 22.2 mM (a, c) and 5.6 mM glucose (b, d). * Statistically significant difference between the (p)ppGpp0 mutant and the (p)ppGpp+ strain in terms of indole and cAMP production (black and blue, respectively) (р ≤ 0.05).

We have previously shown that the effect of (p)ppGpp – a stringent response signaling molecule – in the process of indole production regulation by E. coli cells depends on the glucose content in the cultural medium (Kashevarova et al., 2022). Therefore, (p)ppGpp positively regulates the synthesis of indole under the conditions of its spontaneous formation at high content of glucose in the tryptophan-free medium. However, this did not occur at low glucose concentration. At exogenous addition of 2 mM tryptophan, (p)ppGpp-dependent indole production was observed at both glucose concentrations studied, with a more pronounced effect at the high carbon substrate content. However, glucose limitation at the start of cultivation (in the medium with 5.6 mM glucose) led to an increase in the rate of indole accumulation and a reduction in the time spent to reach its maximum values (Fig. 4).

**Fig. 5.**
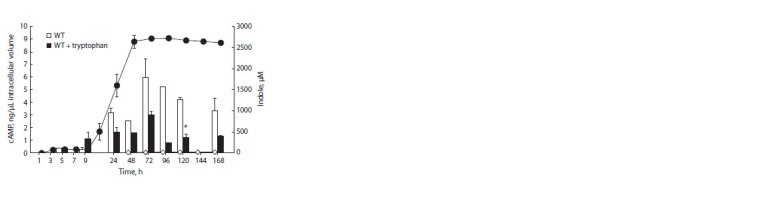
Indole production (graph) and cAMP formation (histogram) in the parent strain in the medium without tryptophan (diamonds/white columns) and with the addition of 2 mM tryptophan (circles/black columns) at 22.2 mM glucose Statistically significant difference between the (p)ppGpp+ strain supplemented with 2 mM tryptophan and the same one grown in the tryptophanfree medium ( р ≤ 0.05).

When studying the effect of indole overproduction on the synthesis of cAMP depending on the carbon substrate content, it was found that the addition of the amino acid tryptophan, which caused the accumulation of indole in the medium to 2.2–2.9 mM (see Fig. 4, c, d ), did not have a significant effect on intracellular cAMP concentration in both parent and deletion strains at the studied glucose concentrations. However, there was an exception: at high glucose content, the (p)ppGpp+ strain demonstrated a decrease in the cAMP levels of up to 4 times compared to the tryptophan-free control in response to tryptophan addition (Fig. 5).

## Discussion

Bacteria, exposed to various stress factors such as starvation, temperature, acid, oxidative stress, etc., have developed effective stress response mechanisms for cell adaptation to unfavorable conditions. Stress responses are induced by signaling molecules that trigger a chain of reactions leading to changes in gene expression and restructuring of cell metabolism, which allows bacteria to survive adverse effects.

In the present work, the mutual influence of the signaling molecules (p)ppGpp, cAMP, and indole upon depletion of glucose in the culture medium was studied. Glucose is the preferred source of carbon and energy for E. coli cells, which have developed a complex regulatory network that coordinates gene expression, transport, and enzyme activity in response to the presence of this substrate (Gutierrez-Ríos et al., 2007). Carbon starvation induces a general stress response regulated by catabolic repression and the (p)ppGpp-mediated stringent response. In E. coli, genes controlled by the catabolic repression regulator protein (CRP) are RelA-dependent, indicating that (p)ppGpp is a global regulator of carbon starvation (Traxler et al., 2006) and demonstrating the interaction between catabolic repression and stringent response mechanisms. A recent study using fluorescence spectroscopy showed that (p)ppGpp is able to bind to the CRP protein with high affinity, and molecular docking results suggested that (p)ppGpp may negatively regulate the activity of this protein. Thus, CRP-controlled gene expression is modulated by (p)ppGpp as a result of its binding to CRP under starvation stress (Duysak et al., 2021).

In bacterial cells, intracellular levels of cAMP are dependent on many factors and, first of all, are determined by the ratio of the enzymes adenylate cyclase and phosphodiesterase activities. The cAMP signaling molecule is a part of the cAMP-CRP complex, which, on the one hand, is controlled by carbohydrates of the phosphoenolpyruvate phosphotransferase system (Deutscher et al., 2006), and, on the other, regulates the catabolite repression of carbon (Botsford, Harman, 1992). The presence of glucose in the growth medium suppresses the expression of enzymes that catalyze the metabolism of other carbon sources, reducing the levels of both CRP protein and cAMP (Ishizuka et al., 1994). In E. coli, cellular cAMP levels are inversely proportional to the concentration of carbon source, resulting in low cAMP content in the presence of an easily metabolized carbon source (glucose). In addition to carbon catabolism, cAMP-CRP regulon controls important cellular functions associated with stress response, cell division, and amino acid metabolism, including the tnaA (tryptophanase) gene, which is responsible for the production of one of the E. coli metabolites, indole, from the amino acid tryptophan (Isaacs et al., 1994).

In this work, the dependence of the dynamics of cAMP and indole synthesis on the presence of (p)ppGpp and glucose contents in the growth medium has been studied. In the experiments, two glucose concentrations of 5.6 and 22.2 mM have been used, which we have designated as low and high glucose contents, respectively. When glucose is depleted in the medium with tryptophan, the metabolism of bacterial cells switches to the utilization of this substrate, which E. coli cells are able to use as the only source of carbon and nitrogen due to the action of the inducible enzyme tryptophanase, which breaks down tryptophan to form indole (Yanofsky et al., 1991).

Our studies have shown that the absence of (p)ppGpp in cells reduced their ability to utilize glucose, which was the most pronounced at high glucose content in the growth medium (22.2 mM). However, an acceleration of glucose consumption from the medium supplemented with tryptophan was demonstrated (see Fig. 1). The ability of bacterial cells to produce (p)ppGpp plays an important role in glucose metabolism, which has been shown for both Gram-positive and Gram-negative bacteria (Oh et al., 2015). In the process of bacterial adaptation to glucose starvation, in addition to stringent response, in which cell growth and the synthesis of macromolecules are inhibited, an expanded adaptive response is formed, including inhibition of glycolysis and metabolic transitions mediated through the mechanism of catabolite repression (Zhang et al., 2016). Our experiments have shown that the depletion of glucose in the medium is accompanied by an increase in the initially low intracellular cAMP content. The maximum level of cAMP accumulation in bacterial cells has been shown to be proportional to the concentration of the initially added substrate. At low glucose concentration (5.6 mM), an increase in the cAMP level occurred earlier than at 22.2 mM glucose (see Fig. 2). At 5.6 mM glucose, the maximum values of intracellular cAMP concentration over a 168-hour period of observation were achieved already at the first day of cultivation in the (p)ppGpp0 mutant and at 24 hours in the parental strain with the addition of tryptophan. At the same time, in the tryptophan-free culture of the (p)ppGpp+ strain, the maximum cAMP level was observed at 72 hours of cultivation (see Fig. 3, a), as well as under the conditions of 22.2 mM glucose addition to the medium (see Fig. 3, b). At initially high glucose content, the level of cAMP was higher in the parental strain as compared to the deletion mutant both in the tryptophan-free medium and in its presence (see Fig. 3, b). Thus, the absence of (p)ppGpp in cells led to a decrease in cAMP formation. These data allow us to assume that the process of cAMP synthesis in E. coli cells is positively regulated by (p)ppGpp.

The second messenger (p)ppGpp is able to modulate gene expression in response to various stress factors in different bacterial species (Irving, Corrigan, 2018). It is a key regulator of stringent response, one of the most important protective mechanisms in bacterial adaptation induced primarily by starvation stress (Traxler et al., 2006, 2008). In Gram-negative bacteria, (p)ppGpp regulates transcription initiation by directly binding to RNA polymerase. In this case, the stimulatory or inhibitory effects of (p)ppGpp on transcription depend on the composition of the discriminatory sequences (AT- or GC-rich) (Sanchez-Vazquez et al., 2019). In addition, (p)ppGpp can directly bind to proteins and thereby change their catalytic activity. Thus, in the work (Ro et al., 2021), it was shown that (p)ppGpp promotes acetylation of the CRP protein, which, as a part of the cAMP-CRP complex, plays a key role in the regulation of gene expression in E. coli. However, there are no significant differences between the parent strain and the mutant one with a deletion of one of two E. coli ΔrelA alarmone synthetases in either the levels of cAMP content or the expression of adenylate cyclase cyaA responsible for the production of cAMP.

Our results have demonstrated the effect of (p)ppGpp on the level of cAMP content, which is displayed in an increase in the concentration of cAMP in the (p)ppGpp+ strain as compared to the mutant strain with the double ΔrelAΔspoT deletion by 2.8 and 6 times at 5.6 and 22.2 mM glucose content, respectively, at 72 hours of growth in the tryptophan-free medium (see Fig. 3). A similar effect of (p)ppGpp on the level of cAMP was also observed under excessive accumulation of indole, but only at high initial glucose concentration in the medium (22.2 mM) (see Fig. 3, b). However, the difference in cAMP content between the WT strain and the knockout one disappeared under glucose limitation (5.6 mM, see Fig. 3, a). The lack of (p)ppGpp effect on cAMP levels (Ro et al., 2021) could probably be explained by the activity of SpoT synthetase leading to the accumulation of (p)ppGpp, while in our experiments the complete absence of stringent response signaling molecule should be ensured by the double ΔrelAΔspoT deletion. Interestingly, the presence of (p)ppGpp in cells accelerated the onset of cAMP synthesis under the conditions of glucose limitation (at the initial content of 5.6 mM glucose in the growth medium), which was observed after 3 hours of growth by a statistically significant 4-fold increase in intracellular cAMP concentration in the (p)ppGpp+ strain as compared to the ΔrelAΔspoT mutant in the tryptophan-free medium. However, at the next two sampling points – 5 and 9 hours of cultivation – the (p) ppGpp0 knockout was superior compared to the parent strain in the level of cAMP production (see Fig. 4, b), which was also displayed under tryptophan supplementation (see Fig. 4, d ).

The present study shows that (p)ppGpp is involved in the regulation of cAMP production, as well as in indole biosynthesis. It is known that the tnaCAB operon responsible for indole biosynthesis is positively regulated by the cAMPCRP- dependent mechanism of catabolite repression and is induced at the transcriptional level upon depletion of carbohydrates and cell transition to the stationary phase (Stewart, Yanofsky, 1985). This explains the observed increase in the cAMP content to levels of 1.8 and 6 ng/μL during cell growth in the tryptophan-free medium at 5.6 and 22.2 mM glucose, respectively (see Fig. 3). This was accompanied by a significant increase in indole formation of up to 10 times at high glucose content in the medium, which is typical only for the parent strain, but not for the mutant one (see Fig. 4, a). Based on these data, we concluded that the alarmone (p)ppGpp along with catabolite repression can positively regulate the process of indole formation. As it follows from Fig. 4, a, b, the intracellular concentration of cAMP was decreased by 6 and 3 times at 22.2 and 5.6 mM glucose, respectively, in the ΔrelAΔspoT mutant as compared to the (p)ppGpp+ strain under growth in the tryptophan-free medium. This indicates a positive role of (p)ppGpp in the regulation of both cAMP synthesis and indole production. Under the addition of tryptophan, both the parent and the mutant strains have demonstrated high indole production at both glucose concentrations studied (see Fig. 4, c, d ). The increase in indole formation occurred earlier in the medium with low glucose content compared to the one with 22.2 mM glucose, which may be due to the earlier formation of cAMP in the cells. At the same time, the mutant has also demonstrated a time delay in indole accumulation, which was more pronounced at high initial glucose content in the medium (see Fig. 4, c). Thus, in E. coli cells, indole production has been shown to be under the positive control of the second messengers (p)ppGpp and cAMP under glucose depletion during batch culture growth.

As shown by our experiments, the cAMP level in E. coli cells depends to some extent on the indole content in the medium. The presence of tryptophan, a substrate for indole synthesis, led to overproduction of indole in the cultures of both the WT strain and the mutant. However, a decrease in cAMP concentration was observed only in the parent strain, which was more pronounced at 22.2 mM glucose (see Fig. 5). However, high indole production in knockout cell culture did not produce a significant effect on the cAMP level.

Therefore, the results obtained in this work demonstrate that the absence of the alarmone (p)ppGpp in E. coli cells led to a decrease in cAMP formation and deceleration in glucose metabolism. An increase in the level of cAMP in response to glucose depletion was accompanied by the synthesis of indole with a pronounced decrease in its accumulation in the (p)ppGpp0 mutant as compared to the parent strain. This was observed in both the tryptophan-free medium and at the tryptophan addition. In cells of the (p)ppGpp+ strain growing in the tryptophan-free medium at a low rate of spontaneous indole formation, its synthesis was significantly increased in response to an increase in the level of cAMP upon depletion of glucose. This phenomenon was not observed in the (p)ppGpp0 mutant that was characterized by a reduced accumulation of cAMP. These data indicate a positive role of (p)ppGpp in the regulation of indole formation, along with the cAMP-mediated catabolic repression. At the addition of tryptophan, both the parental and (p)ppGpp0 strains have demonstrated high indole production, which was accompanied by a decrease in the level of cAMP accumulation in comparison with the tryptophanfree control and was the most pronounced in the (p)ppGpp+ strain at 22.2 mM glucose. Thus, at glucose depletion, the alarmone (p)ppGpp positively regulates the accumulation of cAMP and indole, and the latter, in its turn, reduces the formation of cAMP

## Conclusion

The survival of bacteria in continuously changing environmental conditions is due to a complex, formed during the evolutionary process, system of numerous interacting regulatory networks that control the diverse physiological functions of bacteria under various stress factors. This work studies the interaction of signaling molecules cAMP, (p)ppGpp, and indole, under the conditions of substrate limitation. The results obtained demonstrate that (p)ppGpp functions as a positive regulator of such processes as glucose metabolism, cAMP synthesis, and indole production. The intracellular level of cAMP accumulation depends on both glucose availability in the medium and the stringent response alarmone (p)ppGpp. The absence of (p)ppGpp in cells reduces their ability to produce indole under the conditions of a low rate of its spontaneous formation during cell growth in the tryptophan-free medium and decelerates the rate of indole accumulation in the medium supplemented with tryptophan. Indole biosynthesis in E. coli is positively regulated by the signaling molecules (p)ppGpp and cAMP at substrate limitation. This gives us reason to conclude that (p)ppGpp-dependent indole production is mediated through changes in the level of cAMP in cells. The results obtained demonstrate the modulatory effect of (p) ppGpp on the expression of genes of the tryptophanase operon regulated by means of the cAMP-CRP-dependent mechanism of catabolite repression, the removal of which is induced in response to glucose depletion.

## Conflict of interest

The authors declare no conflict of interest.
